# Abstracts from the 3dMed Australia Conference 2018: Melbourne, Australia. 13-14October, 2018

**DOI:** 10.1186/s41205-018-0036-5

**Published:** 2018-12-18

## Oral Presentations

### O1 - 3D printing: a stepping stone to personalised medicine

#### Jasamine Coles-Black^2^, Jason Chuen^1,2,3^

##### ^1^Department of Vascular Surgery, Austin Health, Heidelberg, Australia; ^2^3dMedLab, Austin Health, The University of Melbourne, Parkville, Australia; ^3^Department of Surgery, The University of Melbourne, Parkville, Australia

###### **Correspondence:** Jasamine Coles-Black (jasaminecb@gmail.com)

*Introduction*: There has been an explosion of interest in 3D printing in Surgery. We seek to demonstrate the contributions to patient-centred care, quality and safety 3D printing brings to current best practice, and break down the perceived barriers to health services adopting this technology.

*Methods*: We performed a literature search using Ovid MEDLINE, Ovid EMBASE and PubMed. The search terms used were “Printing, Three-Dimensional” AND “Surgery”. 392 articles resulted, which were read in full to identify relevant studies. The findings from these studies were then compared against our own centre’s experiences as an early adopter of the technology.

*Results*: We present the four main ways 3D printing is improving quality and safety in Surgery. Firstly, 3D printing allows for improved patient and carer understanding of their anatomy and the planned surgical procedure. 3D printing also improves patient and carer satisfaction and engagement when compared to other media used in the therapeutic relationship. 3D printing has also proven to be a useful adjunct in pre-surgical planning. Patient-specific models have improved patient safety with reduced time under anaesthesia, reduction in operation time, shorter recovery times, and a reduction in blood loss intraoperatively.

In addition, we present solutions to the often-quoted barriers to adoption, such as the lack of technical knowledge, perceived costs, and the lack of definitive regulation in Australia to date.

*Conclusion*: 3D printing lends itself to improving quality and safety by aiding the visualisation of complex anatomy. We seek to highlight the current surgical applications of 3D printing, and the ease with which it can be adopted by health services.

A version of this abstract was previously published as a poster during the Royal Australasian College of Surgeons 87th Annual Scientific Congress [1].

1. ANZ J. Surg. 2018; 88 (S1) 165–69

### O2 - Case series of 3-dimensional printing in spinal fusion. What have we learned thus far?

#### Wen Jie Choy^1,2^, William C. H. Parr^7,8^, Kevin Phan^1,2,3^, Chester E. Sutterlin III^4,5,6^, Ralph J. Mobbs^1,2,3^

##### ^1^NeuroSpine Surgery Research Group (NSURG), Prince of Wales Private Hospital, Sydney, Australia; ^2^Faculty of Medicine, University of New South Wales (UNSW), Sydney, Australia; ^3^Department of Neurosurgery, Prince of Wales Private Hospital, Sydney, Australia; ^4^Department of Neurosurgery, University of Florida, Gainesville, Florida, USA; ^5^ProCRO Pty Ltd, Pyrmont, New South Wales, Australia; ^6^Spinal Health International Inc., Longboat Key, Florida, USA; ^7^3DMorphic Pty Ltd, Sydney, Australia; ^8^SORL, Surgical & Orthopaedic Research Labs, UNSW, Sydney, Australia

###### **Correspondence:** Wen Jie Choy (timanchoy@gmail.com)

*Introduction*: 3D Printing has begun to facilitate spinal surgery in multiple modalities including: pre-operative planning, visualisation of complex anatomy, intra-operative screw positioning, spinal fusion construct design and surgical efficiency. Although relatively uncommon, patients may present with vertebral morphology where conventional “off-the-shelf” implants would provide poor anatomical reconstruction. Utilising 3D printing, custom-made spinal implants (CMSIs) can be designed, printed and implanted into these patients.

*Methods*: We report a single-centre case series of 8 patients who have undergone spinal fusion utilising CMSIs. The indications include 4 malignancies, 2 traumatic injuries, 1 spondylolisthesis and 1 osteoporotic compression fracture.

*Results*: Immediate press-fit was achieved in 7 patients. Surgeon perceived operation time was reduced in all cases. 7 patients had signs of radiological fusion at 3 months post-operation and significant neurological and pain improvement. 1 patient with traumatic injury and poorly mineralised vertebral bone had severe subsidence and non-union accompanied by posterior screw loosening.

*Conclusion*: The CMSIs provide immediate press-fit and early stability with direct ‘mirrored’ surface contact between implant and endplate. This allows more even load distribution throughout the anterior vertebral column and prevents point-loading, which can lead to early subsidence. With 3D printing implant porosity and surface topography can be altered to reduce overall implant stiffness, facilitate osseoconductivity and early bone-device integration. Implant positioning should be considered as the vertebral body is weakest centrally, thus a small, centrally placed implant increases the risk of subsidence.

Bone quality and mineral density of the patient should always be considered as poor bone quality may result in early failure of the construct. Based on the outcomes of our patients, we conclude CMSIs are beneficial for patients with unusual anatomy. However, multiple factors including implant design, implant positioning, surgical approach and patient factors should be carefully pre-planned to achieve a beneficial outcome.

### O3 - Development of photocurable resin for 3D printing of soft and elastic medical devices

#### Timothy Hughes, Johan S. Basuki, Asitha Balachandra, Malsha Wickramaratna, Sean Murphy, Ke Du

##### CSIRO Manufacturing, Clayton, Victoria, Australia

###### **Correspondence:** Timothy Hughes (tim.hughes@csiro.au)

In comparison with conventional manufacturing, additive manufacturing does not require moulds, dies or machining, since the 3D printing technology enables rapid transformation of computer-aided design to complex 3D objects on demand. Although there are different printing platforms available, most commercial polymer 3D printers are useful to fabricate prototypes only. This can be attributed to the lack of biocompatible materials with suitable mechanical properties. Here we report on our efforts to develop new 3D printable resins suitable for Digital Light Projection (DLP) 3D printers for the production of biomedical devices.

In particular, for the fabrication of soft and elastic parts by 3D printing is challenging. For instance, 3D printing of silicone or polysiloxane is very difficult to achieve, although there are needs to have silicone parts prototyped and customized in patient-specific devices. In general, soft materials are prone to plastic deformation during the 3D printing process. Moreover, the resultant 3D elastomeric object does not have sufficient mechanical properties for functional use.

The translation of simple photocure chemistry to a DLP printer is not simple. There are many factors which need to be considering when developing resins for biomedical applications. We have successfully printed soft elastic pasts using an in-house developed siloxane based resin as well as tough and flexible parts aimed at dental applications that exhibit low cytotoxicity and desirable mechanical properties.

### O4 - The benefits of 3D printing in orthopaedic preoperative planning: a literature review

#### Michael Jiang^1,2^, Gordon Chen^1,2^, Jasamine Coles-Black^1^, Jason Chuen^1,2^, Andrew Hardidge^2,3^

##### ^1^3dMedLab, Austin Health, The University of Melbourne, Parkville, Australia; ^2^Department of Surgery, The University of Melbourne, Parkville, Australia; ^3^Department of Orthopaedic Surgery, Austin Health, Heidelberg, Australia

###### **Correspondence:** Michael Jiang (mjiang1@student.unimelb.edu.au)

*Introduction*: 3D printing has seen increasing interest in Surgery, in particular applications where it allows for the visualisation of patient-specific anatomy in complex cases. This literature review investigates the benefits and limitations of 3D printed models in preoperative planning in the field of Orthopaedic Surgery.

*Methods*: A literature search was performed using the Ovid platform on the EMBASE and MEDLINE databases using the terms “3D printing”, “Orthopaedics” and “Surgical Planning”. Studies using 3D printed models as a part of preoperative planning were included. All others were excluded. Data regarding the metrics used to assess the benefit of the use of 3D models, surgical outcome, and surgeon or patient opinion on the technology were extracted.

*Results*: 41 studies resulted. 8 (19.5%) were case control studies, the rest were case reports or case series. Assessment of benefit was mostly subjective, although the case control studies included objective metrics such as operation time, intraoperative blood loss and intraoperative fluoroscopy time. The use of 3D printing technology showed subjective benefit for both patient and surgeon while also indicating promising, clinically significant improvements in intraoperative metrics.

*Conclusion*: Despite the current absence of large scale trials, 3D printing has clear benefits in preoperative planning, particularly when utilised in complex cases. A streamlined workflow for case selection, in-house model creation and pre-operative rehearsals is still required to be developed before the process is ready for routine use.

### O5 - Simulation training using 3D printing in otolaryngology

#### Gordon Chen^1,2^, Michael Jiang^1,2^, Jasamine Coles-Black^1^, Kristy Mansour^4^, Jason Chuen^1,2^, Deborah Amott^2,3^

##### ^1^3dMedLab, Austin Health, The University of Melbourne, Parkville, Australia; ^2^Department of Surgery, The University of Melbourne, Parkville, Australia; ^3^Department of Otolaryngology, Austin Health, Heidelberg, Australia; ^4^Royal Melbourne Hospital, Parkville, Australia

###### **Correspondence:** Gordon Chen (cheng1@student.unimelb.edu.au)

*Introduction*: 3D printing has already proven itself to be a valuable tool in simulation and training in Surgery. We present a literature review of the benefits and limitations of 3D printed surgical simulators in Otolaryngology, one of the most enthusiastic adopters thus far.

*Methods*: A literature search of the MEDLINE and Embase databases was performed using the terms “3D printing,” “Otolaryngology” and “Simulation.” Publications that met inclusion criteria of 3D printed models for the purpose for surgical education were appraised.

*Results:* 23 papers were identified for inclusion (3 of which were conference abstracts). Of these articles, 6 (26%) were prospective cohort studies. The cohort studies objectively measured surgical skill and demonstrated varying levels of construct validity (septoplasty model, endoscopic sinus surgery model, transcanal endoscopic ear surgery model, otosclerosis prosthesis model, costal cartilage airway grafting model). The other 14 (61%) were cross-sectional studies with subjective measures demonstrating largely positive feedback in anatomical fidelity, haptic feedback and value in translation of surgical skill to operating theatre. Several studies reported unanimous interest for integration into curriculum.

*Conclusion*: Though still nascent, 3D printing has already proven to be a valuable component in the surgical education of otolaryngologic trainees across the world. As models become more refined and the barriers to 3D printing lowered, its uses in surgical simulation will continue to expand and become commonplace in surgical skills acquisition.

### O7 - Evolution of computer-assisted surgery and custom-made implants in maxillo-mandibular reconstruction

#### Felix Sim^1,2,3^ (fwsim@bigpond.com)

##### ^1^Royal Melbourne Hospital, Parkville, Victoria, Australia; ^2^Monash Health, Clayton, Victoria, Australia; ^3^Barwon Health, Geelong, Victoria, Australia

*Introduction*: The purpose of this investigation is to critically examine the evolution of this practice, reconstructive outcomes and implementation of this technology in a universal health care system with budget constraints.

*Methods*: Retrospective case series of patients with a variety of benign and malignant tumors who were managed utilising a combination of CAD/CAM software for presurgical planning, stereolithographic models, and intraoperative navigation. CT scan data was obtained in all patients, providing a 3-dimensional rendering of the head and neck for purposes of visualisation, orientation, and diagnosis. The images were analysed with 2D and 3D linear and volumetric measurements and were virtually manipulated (surgical simulation) by mirroring, segmentation, or insertion of anatomic structures with the aid of a software engineer. Post treatment outcome was assessed with CT scan and compared to the virtual plan for accuracy

*Results*: Patient specific cutting guides improved accuracy and ease for reconstruction with fibula free flaps, especially in multi-segment reconstruction. The introduction of patient specific custom plates did not show a clinically significant improvement in accuracy of bone position when compared to standard prebent stock reconstruction plates.

*Conclusion*: Virtual Surgical Planning (VSP) and 3D printing has resulted in an efficient and accurate method of optimizing functional and esthetic maxillofacial reconstructive outcomes. Patient specific fixation hardware do not offer improved accuracy and should only be used in select cases.

### O8 - Three-dimensional pathology specimen modeling using “structure-from-motion” photogrammetry

#### Shane Battye^1,2^ (s.battye@gmail.com)

##### ^1^Histolab Pty Ltd, Kew, Victoria, Australia; ^2^Pathobin Pty Ltd, Balwyn North, Victoria, Australia

*Introduction*: Structure-from-Motion (SfM) photogrammetry is a method of rapid, automated, image-based modelling in which data points in digital images, taken from offset viewpoints, are analysed to generate a 3D model. This modelling technique has been widely used in the context of geomorphology, architecture and digital archiving of artistic sculptures; but has yet to be used within the realm of anatomic pathology.

The objective was to describe the application of a purpose built SfM system in a pathology lab, capable of producing high-quality 3D digital models and its uses in routine surgical pathology practice as well as medical education.

*Methods*: Using custom interface software, Pathoscan (Pathobin) which incorporated the Canon EOS SDK (Canon) and commercial SfM software, Photoscan (Agisoft); we modelled specimens from participating laboratories. The Pathoscan software automates the photogrammetry process with a Canon EOS camera and Pathobin digital turntable, to generate the 3D model.

*Results:* The entity demonstrated in each specimen was well demarcated and easily identified; with the default output of the system in a small 3D PDF file, typically less than 10mb, it can be viewed and manipulated within Adobe Reader (Adobe Systems, USA). More advanced viewing and manipulation techniques were achieved by exporting the model in Wavefront Object (OBJ) format with animations applied in Blender graphics software (Blender Foundation) or exported for colour sandstone printing. Additionally, models were imported into virtual environments using Unity (Unity Technologies) and viewed through virtual reality headsets.

*Conclusion*: Macroscopic 3D modelling of specimens can be achieved through Structure-from-Motion photogrammetry technology and applied quickly and easily in routine laboratory practice and educational settings. 3D modelling in pathology improves clinic-pathologic correlation over traditional photos and enhances medical education, revolutionizing the digital pathology museum with virtual reality environments and 3D printed specimen models.

### O9 - Can we print 3D diamond?

#### Kate Fox^1^, Phong Tran^4^, Aaqil Rifai^1^, Avik Sarker^1^, Andrew Greentree^3^, Nhiem Tran^2^

##### ^1^School of Engineering, RMIT University, Melbourne, Victoria, Australia; ^2^School of Science, RMIT University, Melbourne, Victoria, Australia; ^3^ARC Centre of Excellence for Nanoscale BioPhotonics, School of Science, RMIT University, Melbourne, Victoria, Australia; ^4^Institute of Health and Biomedical Innovation, Queensland University of Technology, Kelvin Grove, Queensland, Australia

###### **Correspondence:** Kate Fox (kate.fox@rmit.edu.au)

Additive manufacturing is presenting new opportunities for medical devices and in particular implants. As the opportunities rapidly increase, researchers are striving to increase the available materials and improve the interface that the implants are providing with underlying bone. Recently we have shown that as printed SLM titanium implants can be built to improve the implant-hard tissue interface suggesting that the interface itself can be influenced by the SLM titanium printing parameters (1). Here we will also discuss recent bench top progress in making diamond-based implants for hard tissue and soft tissue interaction. Diamond, being an analogue of carbon presents a biomaterial well accepted by the body as evidenced by its use in medical bionics implants such as the Bionic Eye. Both the hard tissue (polycrystalline diamond-titanium) and soft tissue (nanodiamond-polycaprolactone) implants show superior biocompatibility and antimicrobial effects than the base material (2).

References

1. Sarker, A, Tran, N, Rifa, i A, Elambasseril, J, Brandt, M, Williams, R, Leary, M and Fox, K. Angle defines attachment: Switching the biological response to titanium interfaces by modifying the inclination angle during selective laser melting, Materials and Design 2018, 10.1016/j.matdes.2018.05.043

2. Rifai, A, Tran, N, Lau, D, Elbourne, A, Zhan, H, Stacey, A, Mayes, E, Sarker, A, Ivanova, E, Crawford, R, Tran, P, Gibson, B, Greentree, A, Pirogova, E and Fox, K. Polycrystalline Diamond Coating of Additively Manufactured Titanium for Biomedical Applications, ACS Appl. Mater. Interfaces 2018, 10, 8474−8484

### O10 - Biofabrication of human articular cartilage: a path towards the development of a clinical treatment

#### Carmine Onofrillo^1,3^, Serena Duchi^1,2^, Cathal O'Connell^2^, Romane Blanchard^1^, Peter Choong^1,2,3^, Gordon Wallace^2^, Claudia Di Bella^1,2,3^

##### ^1^Department of Surgery, The University of Melbourne, Parkville, Australia; ^2^ARC Centre of Excellence for Electromaterials Science, Intelligent Polymer Research Institute, The University of Wollongong, Wollongong, Australia; ^3^Department of Orthopaedics, St Vincent’s Hospital Melbourne, Melbourne, Australia

###### **Correspondence:** Carmine Onofrillo (carmine.onofrillo@unimelb.edu.au)

*Introduction*: Cartilage injuries cause pain and loss of function, and if severe may result in Osteoarthritis. 3D bioprinting is a tangible option for the delivery of bioscaffolds capable of regenerating cartilage tissue. We have developed a handheld device, the Biopen, to allow in situ additive manufacturing during surgery. Given its ability to extrude in a core/shell manner, the Biopen can preserve cell viability during the biofabrication process, and it is currently the only biofabrication tool tested as a surgical instrument in a sheep model using homologous stem cells. As a necessary step toward the development of a clinically relevant protocol, we aimed to demonstrate that our handheld extrusion device can successfully be used for the biofabrication of human cartilage.

*Methods*: We specifically used human derived mesenchymal stem cells (hADSCs), harvested from the Infra-Patellar Fat Pad of patients affected by Osteoarthritis, envisioning them as the source of cells for future clinical applications. With the Biopen, we generated bioscaffolds made of hADSCs laden in Gelatin Methacrylate (GelMa), hyaluronic acid methacrylate (HAMa) and cultured in chondrogenic stimuli for eight weeks in vitro. A comprehensive characterisation including gene and protein expression analyses, immunohistology, confocal microscopy, second harmonic generation, light sheet imaging, and mechanical unconfined compression was carried out.

*Results*: We obtained hyaline-like cartilage formation as demonstrated by accumulation of Collagen type II transcript and protein. The accumulation of Collagen type II is combined with the organization of mature collagen fibrils that provides the strength and the function to the new tissue, which display a Young Modulus of 50KPa.

*Conclusion*: In this study, we have demonstrated that hADSC can produce hyaline-like cartilage in GelMa/HAMa bioscaffolds. The in vitro biofabrication of human neocartilage via a handheld extrusion device is a key step in the path towards the development of tissue regeneration strategies in clinical practice.

### O11 - Assessment of coronary artery obstruction risk during transcatheter aortic valve replacement utilising 3D printing

#### Jeremy Russo, Ronen Gurvitch and Tamara Yuen

##### Department of Cardiology, Royal Melbourne Hospital, Melbourne, Australia

###### **Correspondence:** Jeremy Russo (russo.jeremy93@gmail.com)

*Introduction*: In patients at high risk of coronary artery obstruction (CAO) during transcatheter aortic valve replacement (TAVR), current imaging techniques are inadequate to rule out this fatal complication. Advancements in 3D printing may allow the development of models capable of replicating cardiac anatomy and predicting CAO. We sought to utilise this technology to improve CAO risk assessment through TAVR simulation and overcome uncertainties with procedural safety.

*Methods*: Seven patients with severe aortic stenosis at high risk of CAO during TAVR were selected for 3D printed modelling. The aortic root, leaflets and sinus, calcium deposits and coronary ostia were precisely reconstructed with Materialise HeartPrint Flex technology from CT imaging. The patency of the coronary ostia was assessed for obstruction after a valve prosthesis was appropriately sized and deployed in each 3D model.

*Results*: Model-derived simulation results were compared to clinical outcomes. Two high-risk TAVR cases were abandoned following transient CAO during balloon aortic valvuloplasty (BAV). Following BAV in case one, a large aortic leaflet calcification clearly obstructed the left main coronary ostium. During 3D model simulation the calcified leaflet was deflected into the left main ostium obstructing the opening, correlating with clinical circumstances. In case two, the heavily calcified aortic leaflet rose over the left main ostium during BAV, abutting the sinotubular junction. This was evident during simulation with the left main ostium 100% obstructed by the calcified left valve leaflet. High risk cases that were clinically uncomplicated during TAVR also did not obstruct during model simulation.

*Conclusion:* In this proof of concept study, we have demonstrated that model-derived TAVR simulation results correlated with clinical outcomes. 3D printed cardiac models of patients at high risk of CAO may be utilised in pre-procedural planning to accurately predict this complication. With development, 3D models may contribute to minimising procedural complications leading to safer patient outcomes.

### O12 - Crime in 21st century: application of 3d methods in forensic medicine

#### Agnieszka Sekula^1,2^, Michael J. Thali^2^

##### ^1^Laboratory for Translational and Molecular Imaging, Duke-NUS Medical School, Singapore, Singapore; ^2^Institute of Forensic Medicine, University of Zurich, Zurich, Switzerland

###### **Correspondence:** Agnieszka Sekula (sekulaagnieszka@gmail.com)

*Introduction*: Advancements in 3D methods, employed at the Institute of Forensic Medicine at University of Zurich (IRM-UZH), resulted in the founding of a dedicated 3D Centre. Collaboration between the IRMUZH, the Forensic Institute Zurich, City Police Zurich and Canton Police Zurich now delivers professional services in detection, documentation, simulation and reconstruction of forensic findings.

*Methods*: Numerous tools were custom developed in service of this practice. Virtobot, an automated, robotic system, combines optical scanning and photogrammetry for surface documentation of deceased bodies. Its integration with segmented CT data yields a comprehensive model of the whole body. Documentation of living individuals as well as relevant objects, including vehicles and weapons, is performed with a mobile version of similar tools. Collected data constitutes 3-dimensional, textured models, which aid forensic investigations. This 3D workflow, leading to fully digitalized and highly accurate reconstructions, is now a common practice in Zurich.

*Results/Conclusion*: This presentation will cover innovative 3D techniques used in forensic practice, highlighting why it is crucial for advancements in the field, and what other professionals can learn from it. A skull reconstruction process, starting from 3D documentation all the way to visualization of injury mechanics, will serve as an example.

Reference

1. Ebert L. et al. Forensic 3D surface documentation at the Institute of Forensic Medicine in Zurich – workflow and communication pipeline; Journal of Forensic Radiology and Imaging, Jun 2016; 5;1-7.

### O13 - Toward prevention of spinal fracture using subject-specific 3D computer models and imaging techniques

#### Hossein Mokhtarzadeh^1,2,3^, Dennis Anderson^1,2^, Mary Bouxsein^1,2^

##### ^1^Department of Orthopedic Surgery, Harvard Medical School, Boston, MA, USA; ^2^Center for Advanced Orthopaedic Studies, Beth Israel Deaconess Medical Center, Boston, MA, USA; ^3^Department of Mechanical Engineering, Melbourne School of Engineering, University of Melbourne, Parkville, Australia

###### **Correspondence:** Hossein Mokhtarzadeh (mokhtarzadeh.hossein@gmail.com)

Spinal fracture and aging are interrelated. Diagnosis of such a challenging incident is quite complex. Currently, imaging techniques (e.g. DEXA scans) are being used to predict the risk of spinal fracture. However, bone mineral density (from imaging) is not a strong indication of future fractures. At Harvard, we have been developing subject-specific 3D computer-based models using QCT data and cutting-edge biomechanical models to predict the location of fracture and type of activities that lead to such debilitating fractures. This presentation will demonstrate some of our recent findings toward prevention of spinal fracture in a large cohort of community-based subjects.

### O14 - Utility of 3D printed models of Müllerian anomalies as a teaching tool

#### Rose Hadden^1^, Sonia Grover^2^, Jason Chuen^1,2,3^, Jasamine Coles-Black^1^

##### ^1^3dMedLab, Austin Health, The University of Melbourne, Parkville, Australia; ^2^Royal Children's Hospital, The University of Melbourne, Parkville, Australia Department of Surgery, Austin Health, The University of Melbourne, Parkville, Australia

###### **Correspondence:** Rose Hadden (rosehadden@gmail.com)

*Introduction*: Congenital Müllerian anomalies can be rare and complex. An inadequate understanding of these anomalies can result in incorrect or suboptimal surgery and unnecessary diagnostics. Alternatively, patients may be required to travel long distances to specialist centres for relatively straight forward procedures. Medical imaging facilitates pre-operative planning, but these modalities tend to present only 2-dimensional representations, limiting optimal conceptualisation of complex anatomy. This may be overcome with 3D models. 3D printing has been successfully used in a number of other disciplines, including Vascular Surgery, Neurosurgery and Cardiac Surgery. The use of 3D printing in Gynaecology has thus far been limited. This project aimed to assess the feasibility of producing 3D models of congenital Müllerian anomalies and to assess their utility.

*Methods*: MRI images of patients with a known Müllerian tract anomaly were collected. The de-identified scans were processed to create a 3D software model, which was then printed at the Austin Health 3dMedLab.

Gynaecologists and trainees attending a paediatric and adolescent Gynaecology educational session were asked to assess the utility of the models utilising a sliding scale survey.

*Results*: Developing the 3D models was found to be challenging and time consuming when compared previous experiences in developing models of vasculature or solid organ structures, where contrast is utilised to highlight the regions of interest.

3D models were found to increase the Gynaecologists’ understanding of these anomalies and, to a lesser extent, their confidence in surgery to correct them. The majority of doctors also responded very positively with regards to the utility of models to enhance patient understanding.

*Conclusion*: Although understanding of the anomaly improved with the 3D models, the capacity to make Müllerian anomaly models from MRI was more challenging than other anatomical regions. Thus while they may be useful for training and patient education, this may limit its use in individual case preparation.

### O15 - 3D CT with a virtual reality interface enhances preoperative planning for complex liver surgery

#### Lawrence Lau^1^, Albert Fung^1^, Paul Kelly^1^, Jean Yi Chun Lin^1^, Joy Qu^1^, Paul Greig^1,2^, Ian McGilvray^1,2^

##### ^1^Toronto Video Atlas of Surgery, Toronto General Hospital, University Health Network, Toronto, Canada; ^2^Division of General Surgery, Department of Surgery, University of Toronto, Toronto, Canada

###### **Correspondence:** Lawrence Lau (thelau@gmail.com)

*Introduction*: The liver has highly variable vascular and biliary anatomy. Liver tumours often have intricate spatial relationships between intrahepatic structures which is not visible on the surface of the liver. Currently, computed tomography (CT) images for complex liver resection are limited to two-dimensional (axial/coronal/sagittal) planes. Surgical planning can be refined using a virtual reality (VR) interface to navigate three-dimensional CT reconstructions.

*Methods*: CT scans were obtained with arterial and venous contrast phases. The scans were exported to the Unity3D game engine through a plugin. This transforms CT DICOM data into 3D volumetric renderings. Within Unity3D, the viewing window is adjusted to isolate structures such as solid organs (liver parenchyma), vasculature (hepatic/portal veins, hepatic arteries), as well as pathologic structures (tumours).

Installation of another plugin module in Unity3D allows for the three-dimensional, volumetric structures to be viewed and manipulated (repositioned, rotated, resized) through input from a VR headset (Oculus Rift with Touch controllers).

*Results/Conclusion*: Isolation of the various structures enables the use of visualisation techniques such as transparency and overlays to further improve pre-operative mapping of anatomy in a three-dimensional space. The VR manipulation of the three-dimensional images enables the surgeon to visualise liver anatomy as it would be encountered intra-operatively.

Preoperative appreciation of complex intrahepatic anatomy is enhanced with the VR interface.

### O18 - 3D printed AAA models for pre-surgical simulation

#### Jason Chuen^1,2,3^, Jasamine Coles-Black^2^, Andrew Woo^2^, Chiu Kang^3^, Jason Toniolo^3^, Nathaniel Chiang^1,3^, Mayo Theivendran^1,3^

##### ^1^Department of Vascular Surgery, Austin Health, Heidelberg, Australia; ^2^3dMedLab, Austin Health, The University of Melbourne, Parkville, Australia; ^3^Department of Surgery, The University of Melbourne, Parkville, Australia

###### **Correspondence:** Jason Chuen (jchuen@unimelb.edu.au)

As endovascular aortic aneurysm repair (EVAR) becomes progressively complex, vascular surgeons are finding the need for representative preoperative visualisation increasingly critical to surgical decision-making and intraoperative performance. The variety of open and endovascular surgical options, ranging from standard, chimney, fenestrated and branched grafting means that detailed surgical planning is essential.

In our case series over one year, computer workstation review after 3D segmentation and STL modelling of abdominal aortic aneurysms (AAA) has significantly influenced surgical decision making and device selection. In addition, these models have been successfully 3D printed and demonstrated to be useful in a variety of settings, including patient education and engagement, surgical and anatomical education, as well as intraoperative visualisation.

These models can be manipulated further to generate an entire hollow 3D printed thoracoabdominal aorta with branches which, despite material limitations, successfully mimic the cannulation and deployment challenges encountered during live endovascular surgery. Further gains are expected as dimensional and representational material validity is improved.

### O20 - Spinal BioModelling: my experience of 680 cases from 1994-2018

#### Paul D’Urso (paul@anatomics.com)

##### Anatomics Pty Ltd, St Kilda, Victoria, Australia

The unique bony, neural and vascular anatomy of the spine demands meticulous preoperative planning and surgical technique to achieve safe and effective instrumented fusion surgery. Additive manufacturing methods provide surgeons with the opportunity to develop BioModels, surgical guides, retractors and custom titanium implants to improve surgical outcomes.

The author presents his personal experience in 680 cases where surgeon-led planning, patient CT data, software simulation and additive manufacturing techniques were combined to develop patient-specific implants, drilling templates, and stereotactic spinal BioModels for the planning, informed consent of patients, and the execution of spinal surgery.

In all cases spinal BioModelling simplified the surgery. BioModels, templates and guides facilitated intraoperative navigation and positioning of instrumentation using standard fluoroscopy. 3D printed titanium implants fitted easily and removed the need to fashion suitable implants intraoperatively. Radiographic follow-up in all cases demonstrated anatomical restoration with <1% of hardware complications.

These cases demonstrate the feasibility of developing patient-specific 3D printed BioModels, stereotactic guides, retractors and custom implants by combining preoperative surgical planning with biomodelling and 3D printing to simplify spinal instrumentation surgery.

